# Differential regulation of the unfolded protein response in outbred deer mice and susceptibility to metabolic disease

**DOI:** 10.1242/dmm.037242

**Published:** 2019-02-27

**Authors:** Amanda Havighorst, Youwen Zhang, Elena Farmaki, Vimala Kaza, Ioulia Chatzistamou, Hippokratis Kiaris

**Affiliations:** 1 Department of Drug Discovery and Biomedical Sciences, College of Pharmacy, University of South Carolina, Columbia, SC 29208-3402, USA; 2 Peromyscus Genetic Stock Center, University of South Carolina, Columbia, SC 29208-3402, USA; 3 Department of Pathology, Microbiology and Immunology, School of Medicine, University of South Carolina, Columbia, SC 29208-3402, USA

**Keywords:** ER stress, Expression profile, Prediction, Lipidemia

## Abstract

Endoplasmic reticulum (ER) stress has been causatively linked to the onset of various pathologies. However, whether and how inherent variations in the resulting unfolded protein response (UPR) affect predisposition to ER-stress-associated metabolic conditions remains to be established. By using genetically diverse deer mice (*Peromyscus maniculatus*) as a model, we show that the profile of tunicamycin-induced UPR in fibroblasts isolated at puberty varies between individuals and predicts deregulation of lipid metabolism and diet-induced hepatic steatosis later in life. Among the different UPR targets tested, *CHOP* (also known as *Ddit3*) more consistently predicted elevated plasma cholesterol and hepatic steatosis. Compared with baseline levels or inducibility, the maximal intensity of the UPR following stimulation best predicts the onset of pathology. Differences in the expression profile of the UPR recorded in cells from different populations of deer mice correlate with the varying response to ER stress in altitude adaptation. Our data suggest that the response to ER stress in cultured cells varies among individuals, and its profile early in life might predict the onset of ER-stress-associated disease in the elderly.

This article has an associated First Person interview with the first author of the paper.

## Abstract (precis)

**Summary:** By using genetically diverse deer mice, we show that the expression of different chaperones is highly coordinated in individual animals and its profile predicts the onset of metabolic pathology.

## INTRODUCTION

The identification of factors that can predict the development and outcome of metabolic health conditions later in life is of paramount significance. The former can be used to identify individuals at increased risk of disease and provide molecular targets for therapeutic intervention. To this end, the study of functional tests that reflect the efficiency of converging biochemical pathways that are involved in disease may have an advantage over examining specific disease-linked polymorphisms. Although such polymorphisms can reveal malfunction of designated pathways, they have limited power in estimating the subsequent development of multifactorial pathologic states.

An array of diseases, especially those affecting the elderly, such as diabetes (Back and Kaufman, 2012), hepatic steatosis (Han and Kaufman, 2016), neurodegenerative diseases (Hetz and Saxena, 2017) and others, have been causatively linked to endoplasmic reticulum (ER) stress and the deregulation of the resulting unfolded protein response (UPR) (Frakes and Dillin, 2017; Zhu and Lee, 2015). In the context of liver dysfunction and hepatic steatosis, research has shown that loss of function of particular UPR targets may result in the onset of steatosis, underscoring the potential protective role of the UPR in disease development (Yamamoto et al., 2010; Ji et al., 2011; Chen et al., 2014a,b; Kammoun et al., 2009). Alternatively, other investigations identified the pro-lipogenic role of certain UPR targets such as *CHOP* (also known as *Ddit3*) (Rutkowski et al., 2008), underscoring the complex integration of the various components of the UPR in the development of steatosis. Although such approaches clearly establish correlation between specific UPR genes and disease, they suffer from the fact that genetic ablation of a single locus may be biologically irrelevant and can trigger phenotypes that are qualitatively distinct from those stemming naturally from hypomorphic versions of UPR-associated genes. Indeed, it is not uncommon for different quantitative inputs to provide distinct qualitative outputs, thus complicating data interpretation and negatively impacting meaningful and clinically relevant conclusions (Garcia and Phillips, 2011). In addition, experimentation and disease modeling in genetically homogenous, inbred mice, although a superior choice for mechanistic studies, is inadequate in addressing questions regarding the regulation of ER stress response in diverse populations that potentially exhibit varying levels of UPR among individuals (Wade and Daly, 2005). For example, although the UPR is a rather global response that involves the simultaneous activation of many different components, its deregulation is usually studied at the level of overexpression of specific chaperones upon exposure to the stress stimulus. Such an approach ignores whether, aside from expression levels of a single target, its relative levels compared with other UPR targets are also associated with pathogenicity (Hetz et al., 2013; Gardner et al., 2013). Thus, studying the profile of the UPR in the context of inherent variability between individuals may bear value in predicting predisposition to ER-stress-associated diseases and could also provide unforeseen clues regarding disease pathogenesis.

A potential limitation in addressing these questions is related to the lack of adequate animal models beyond conventional laboratory mice (*Mus*). The latter are inbred, or derivatives of inbred strains, and therefore exhibit limited genetic diversity. Unless specific mutations are being studied, the use of mice does not allow for assessment of varying UPR or appreciation of its impact in disease development. In order to overcome these issues and explore the outcomes of varying UPR in naturally existing populations, we used outbred deer mice as a model (*Peromyscus maniculatus*) (Havighorst et al., 2017; Bedford and Hoekstra, 2015). In this genetically diverse system, naturally existing quantitative variation in the UPR, rather than the qualitative changes typically induced in mice – such as genetic deletion or forced overexpression of specific genes of interest – could be studied more effectively.

## RESULTS

### The profile of the UPR varies in fibroblast cultures of deer mice

**Fig. 1.**
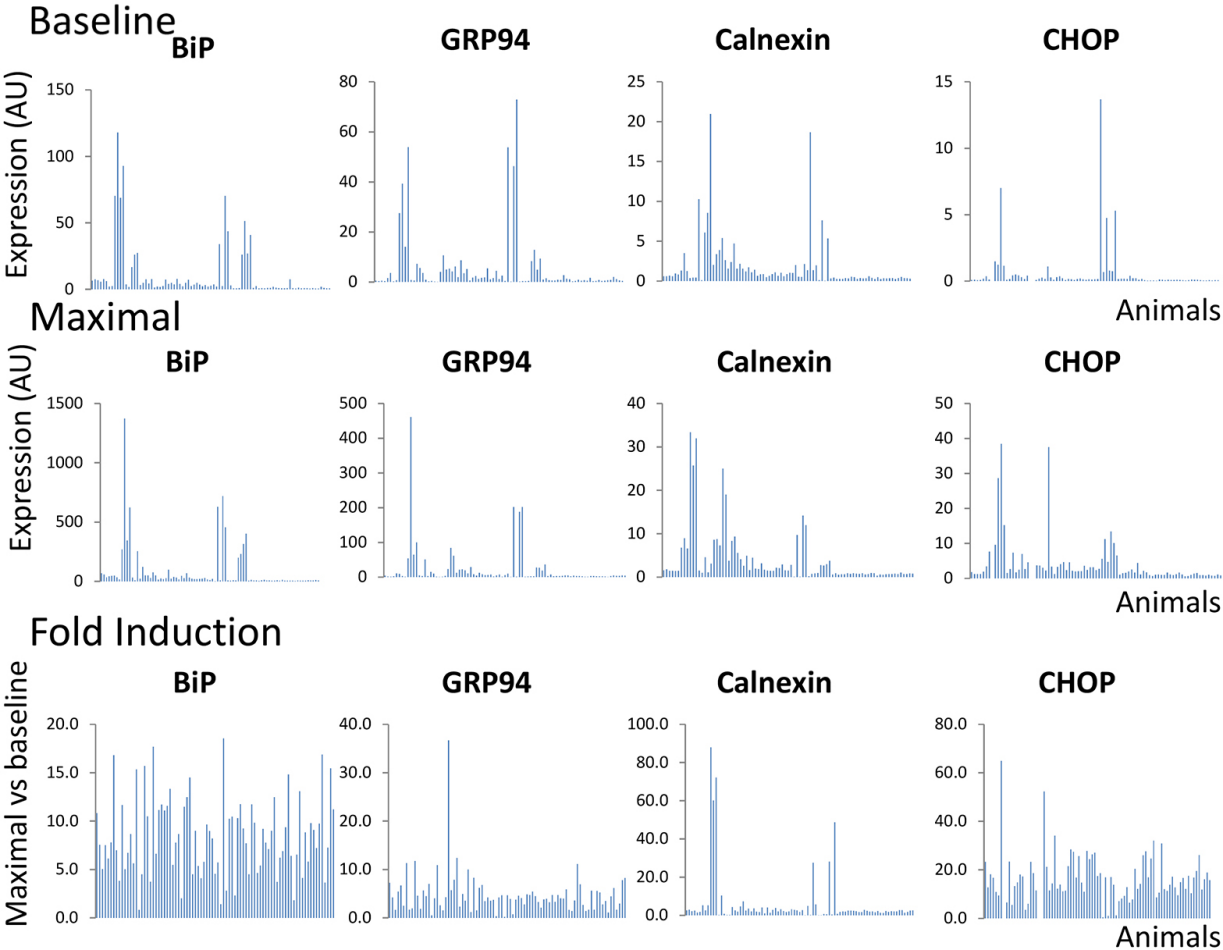
**Expression of *BiP*, *GRP94*, calnexin and *CHOP* in primary fibroblasts isolated at puberty from *P. maniculatus*.** Expression prior to (baseline) and after (maximal) exposure to tunicamycin (5 µg/ml) for 5 h, and the ratio of maximal versus baseline expression (fold induction), is shown. All expression values were normalized in relation to *Gapdh* (*n*=85; 43 males and 42 females). Each bar represents a different animal.

**Fig. 2.**
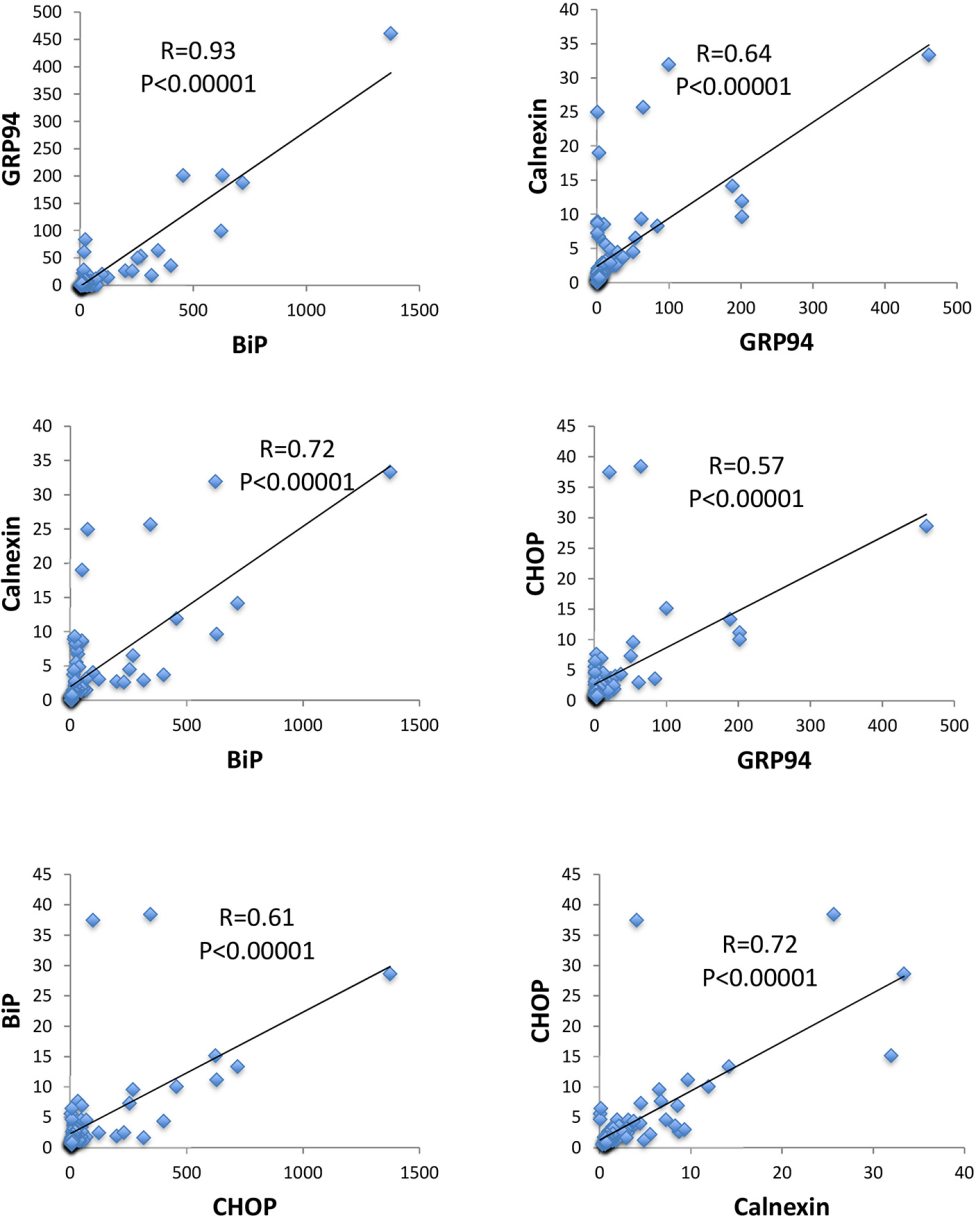
**Coordinated expression of UPR-associated genes in primary fibroblasts of *P. maniculatus*.** Pairwise comparisons in expression (arbitrary units) of ER-stress-related genes after exposure of cells to tunicamycin. R-values from Pearson's correlation and *P*-values are shown. All expression values were normalized in relation to *Gapdh* (*n*=85; 43 males and 42 females).

Initially, we sought to explore whether and to what extent the UPR profile differs among individual animals. To address this, primary fibroblast cultures (<4 passages) were established during puberty from a genetically diverse population of *P. maniculatus* (*n*=85; 43 males and 42 females) and the UPR profile was assessed after exposure of cultured cells to 5 µg/ml tunicamycin for 5 h. As shown in Fig. 1, high variation in the UPR profile was detected, as monitored by the baseline levels (top row), fold induction levels following tunicamycin exposure (bottom row) and maximal levels (middle row) of a roster of UPR-associated genes that included chaperones *BiP* (also known as *Hspa5*), *GRP94* (also known as *Hsp90b1*) and calnexin, and the ER-stress-associated transcription factor *CHOP*. Because tunicamycin is a rather global activator of ER stress and an inducer of the UPR (Li and Lee, 1991), we expected to record a positive correlation in the expression of different UPR-related genes in the same individuals. Indeed, a strong positive correlation was identified for all pairwise comparisons (*P*≤0.0001, Pearson's) when maximal levels of expression in tunicamycin-treated cells were considered (Fig. 2). Those included (clockwise from top left in Fig. 2) *BiP* versus *GRP94*, *GRP94* versus calnexin, *GRP94* versus *CHOP*, calnexin versus *CHOP*, *CHOP* versus *BiP*, and *BiP* versus calnexin. However, when either the baseline levels of expression of untreated cells or fold induction (defined as the ratio of maximal to baseline) after tunicamycin treatment were analyzed, correlation was decreased and reached significance only in specific pairwise comparisons. Therefore, we concluded that the maximal levels of UPR-associated gene expression likely give the most information in terms of UPR induction, and that the intensity of the UPR ultimately reflects a generalized systemic response that transcends different branches of the UPR rather than a response of specific UPR targets. The systemic nature of this response is also supported by the observation that the expression profile of UPR genes in primary fibroblasts from different animals was similar in thapsigargin- and tunicamycin-treated cells, despite that they induce ER stress by alternate mechanisms (Fig. S1). In line with this is the fact that independent preparations of fibroblasts from the left or the right ear rendered similar results for *GRP94*, *BiP* and calnexin (Fig. S2). Some variation was detected for *CHOP*, which, however, was insufficient to alter considerably the overall magnitude of the response when assessed in comparison with the whole group of animals tested. This variation in *CHOP* is probably related to the fact that *CHOP* is pro-apoptotic and therefore likely subjected to strong selective pressure during the culture of primary cells (Fig. S2). Noteworthily, this responsiveness of fibroblasts to tunicamycin was progressively abolished because at later passage the inducibility decreased (Fig. S3).

Comparison between males and females showed no significant difference in the profile of the UPR. However, a trend for higher intensity was detected in the former. In males, the expression of at least 2 UPR genes fell in the top 25th percentile in 16 of 43 specimens, compared with 10 of 42 specimens in females (*P*=0.15). These results are consistent with previous studies that demonstrate the protective effect of estrogen signaling against ER stress (Andruska et al., 2015; Kooptiwut et al., 2014).

**Fig. 3.**
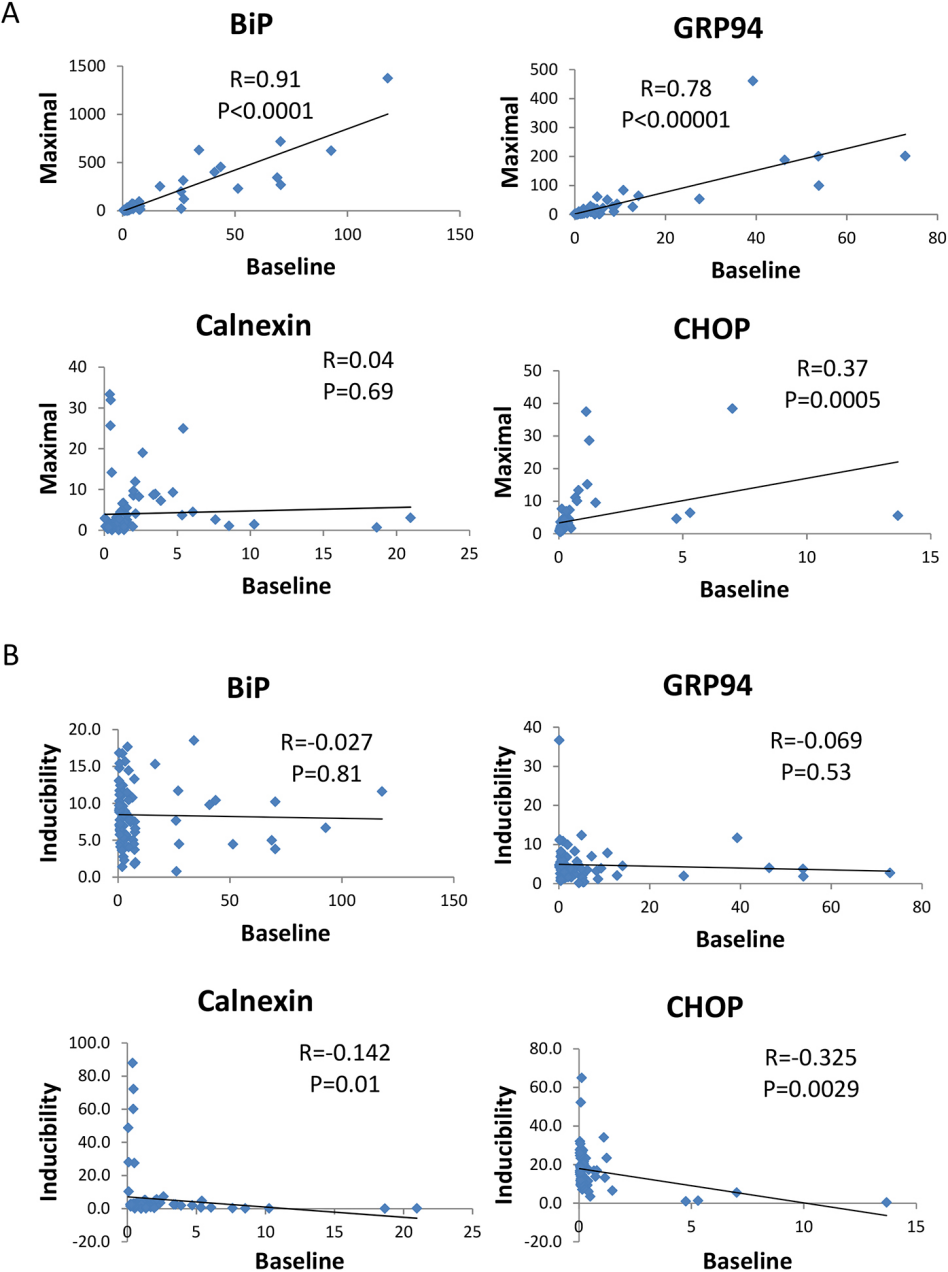
**Pairwise comparisons between the baseline expression versus maximal expression, and baseline expression versus inducibility, for *BiP*, *GRP94*, calnexin and *CHOP*.** (A) Baseline expression versus maximal expression. (B) Baseline expression versus inducibility. R-values and *P*-values from Pearson's correlation are shown. All expression values were normalized in relation to *Gapdh* (*n*=85; 43 males and 42 females).

In order to further investigate the relationship between baseline levels, maximal expression after tunicamycin treatment and levels of induction (ratio of maximal to baseline), we investigated whether maximal and baseline expression were correlated for the different ER stress targets tested. This analysis showed that for *BiP* (*P*<0.0001), *GRP94* (*P*<0.0001) and *CHOP* (*P*=0.0005), but not for calnexin, the maximal levels correlated with the baseline levels (Fig. 3A). In calnexin (*P*=0.01) and *CHOP* (*P*=0.0029), but not *BiP* (*P*=0.81) and *GRP94* (*P*=0.53), a reverse correlation between baseline levels and inducibility was detected (Fig. 3B). These observations imply that, during stress, different UPR genes display a distinct mode of regulation. In some, exemplified by *GRP94* and *BiP*, maximal levels are proportional to the baseline levels, implying the existence of a not highly variable degree of induction. For others, such as calnexin, it appears that inducibility is negatively associated with the baseline expression and positively associated with the maximal levels of expression, indicating that the expression prior to stress determines the extent of stimulation. *CHOP* follows both modes of regulation.

### Plasma lipid levels correlate with the UPR profile of pubertal fibroblasts *ex vivo*

**Fig. 4.**
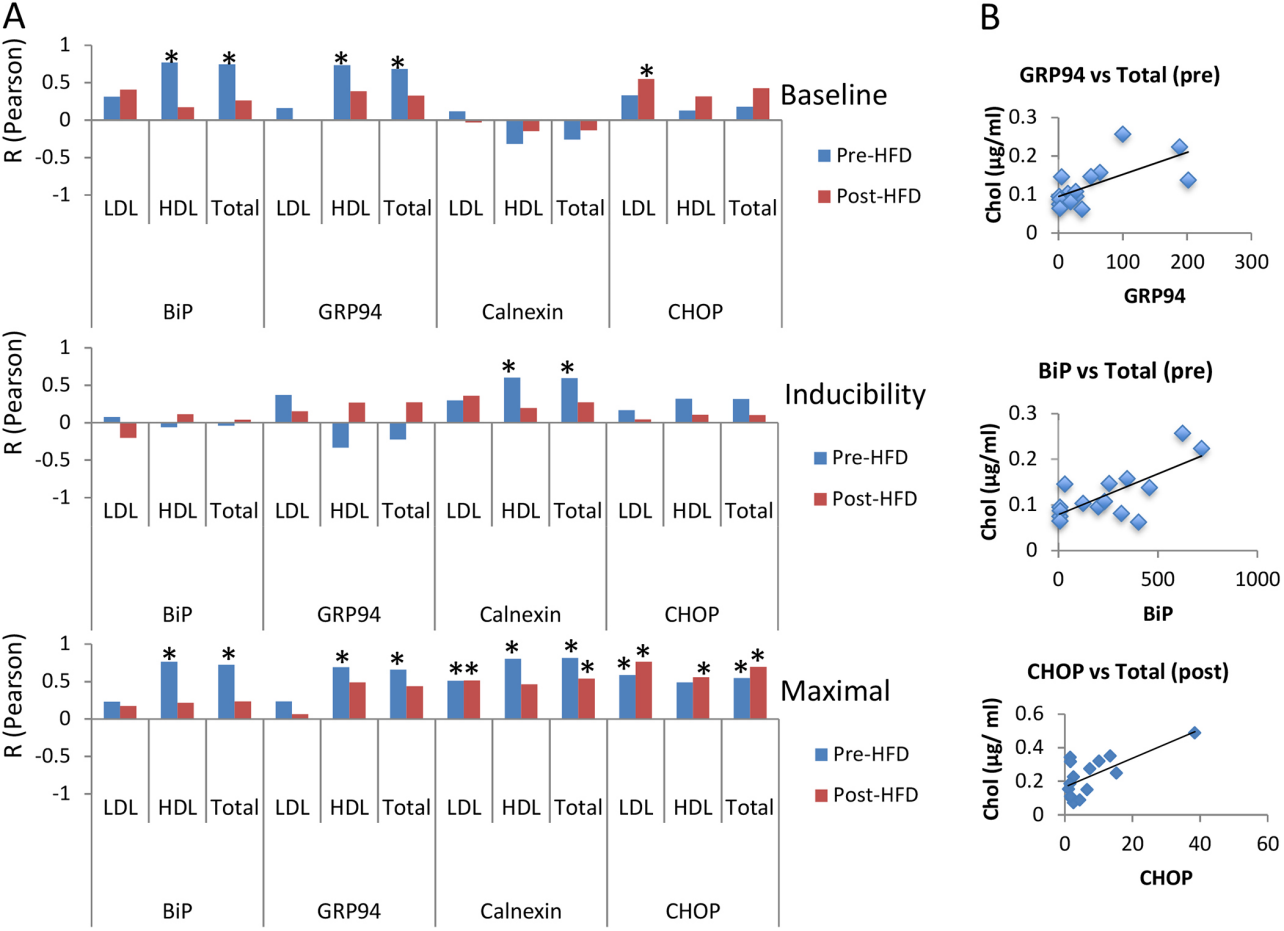
**The UPR profile as recorded in primary fibroblasts is positively correlated with plasma lipid levels.** (A) R-values (from Pearson correlation; *P<0.05) between prior to (baseline) and after (maximal) tunicamycin exposure, and the ratio of maximal versus baseline (inducibility) for *BiP*, *GRP94*, calnexin or *CHOP*, and lipid levels in the plasma of 4- to 5-month-old animals (*n*=15). Lipid levels were assessed in animals receiving regular or high-fat/sucrose diet for 2 weeks. (B) Representative examples of correlation between total cholesterol (Chol) before (pre) or after (post) high-fat-diet (HFD) administration and *BiP*, *GRP94* and *CHOP* in pubertal fibroblasts after exposure to tunicamycin (*P<0.01, Pearson's). HDL, high-density lipoprotein; LDL, low-density lipoprotein.

Next, we wanted to test whether the profile of UPR recorded in fibroblasts *ex vivo* possesses predictive value for the onset of a chronic pathology linked to ER stress. In view of the role of ER stress in the development of metabolic disorders, we studied the association of inherent variation in UPR in primary cultures *ex vivo* with lipid levels prior to or after administration of a high-fat diet. Thus, we measured total cholesterol, low-density lipoprotein (LDL) and high-density lipoprotein (HDL) in the plasma of adult animals aged 4-5 months, before and after short-term (2-week) administration of a high-fat/sucrose diet. We postulated that, during this short time period of high-fat dietary intake, no major histopathological damage would occur in the liver, and therefore the plasma lipid levels would directly reflect the result of lipid metabolism rather than of liver dysfunction. A summary of this analysis is shown in Fig. 4A, and selected examples of correlation between total cholesterol (Chol) before (pre) or after (post) high-fat-diet administration and *BiP*, *GRP94* and *CHOP* are shown in Fig. 4B. Before high-fat-diet administration, all UPR target genes tested showed positive correlation with the lipid levels in the plasma, suggesting that baseline plasma lipid levels directly follow the propensity for individual UPR changes as recorded in primary cell cultures established early in life. When this association with plasma lipid levels was specifically compared with the baseline levels of UPR targets in cultured cells, their maximal levels after tunicamycin exposure or their relative fold induction, the strongest correlation was obtained with the maximal levels of UPR target genes (Fig. 4A). The corresponding baseline levels were correlated with HDL and total cholesterol levels for both *GRP94* and *BiP*. Interestingly, relative calnexin expression in tunicamycin-treated cells was significantly correlated with HDL and total cholesterol levels, but this was not the case with the other tested UPR targets.

**Fig. 5.**
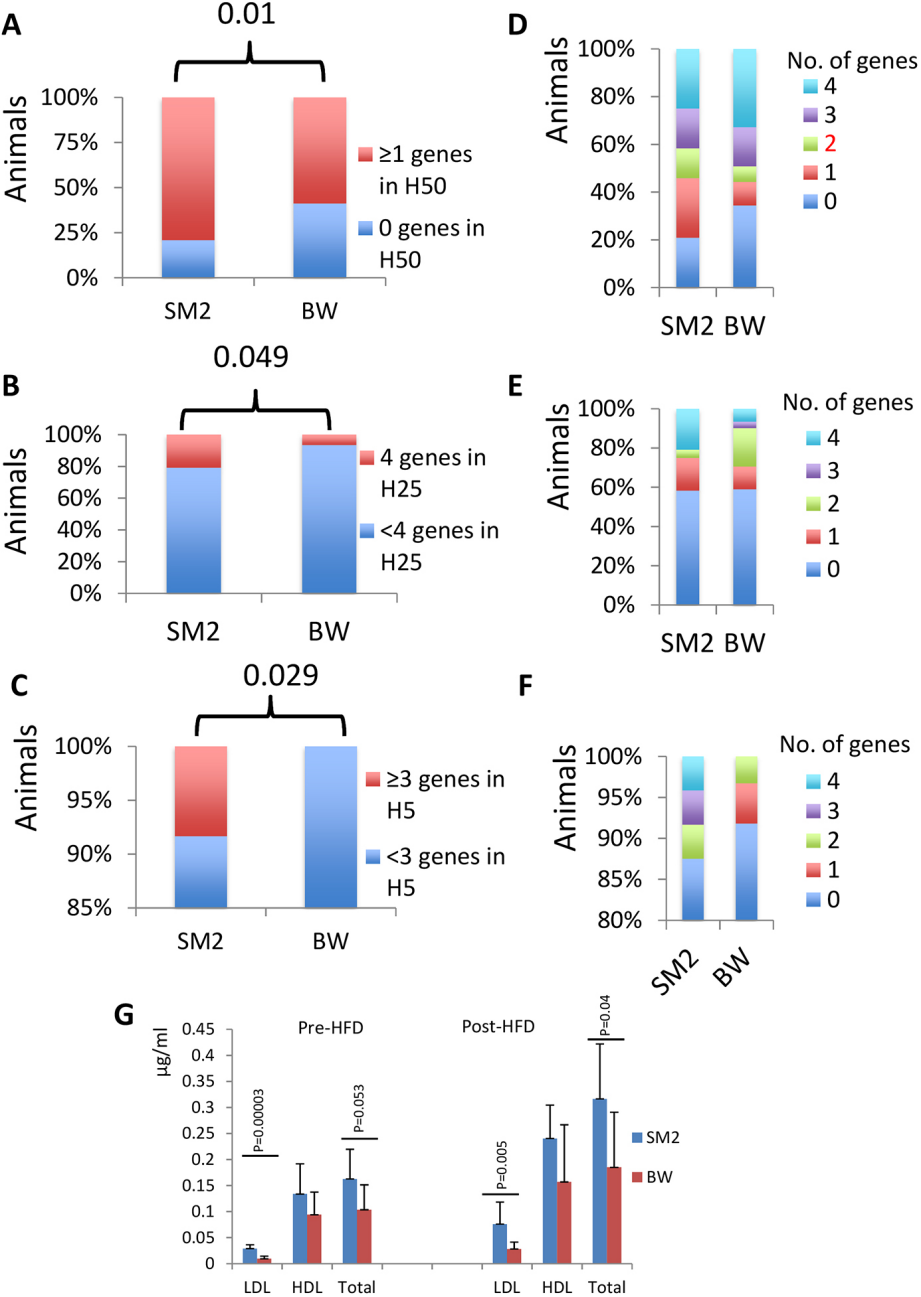
**Differences in the UPR profile in primary fibroblasts between high (SM2 population, *n*=24)- and low (BW population, *n*=61)-altitude deer mice.** (A) Percentage of animals in each of the SM2 or BW populations that have at least 1 UPR gene in the highest 50th percentile (H50) of the total population. The *P*-value is shown (chi-square test). (B) Percentage of animals in each of the SM2 or BW populations that have all 4 UPR genes in the highest 25th percentile (H25) of the total population. The *P*-value is shown (chi-square test). (C) Percentage of animals in each of the SM2 or BW populations that have 3 or 4 UPR genes in the highest 5th percentile (H5) of the total population. The *P*-value is shown (chi-square test). (D-F) The corresponding distribution for the number of genes in each analysis in A, B and C, respectively. For these analyses, maximal expression was considered. (G) Average lipid levels in SM2 (*n*=5) or BW (*n*=10) prior to or after high-fat-diet (HFD) administration for 2 weeks. *P*-values (Student’s *t*-test) are shown. HDL, high-density lipoprotein; LDL, low-density lipoprotein.

In challenged animals that had received a high-fat diet for 2 weeks, plasma lipid levels increased (Fig. 5G), but *BiP* levels in culture ceased to be associated with lipid levels in the plasma as they were prior to diet-induced challenge (Fig. 4). However, the maximal levels of *CHOP* and calnexin levels continued – with the exception of calnexin and HDL – to show association with the plasma lipid levels in the same way as they did prior to high-fat-diet administration. It is possible that, while BiP is mostly associated with basal lipid metabolism, under conditions of metabolic challenge, CHOP and calnexin are engaged more. A role in promoting lipid accumulation has been demonstrated for CHOP (Rutkowski et al., 2008); however, we are unaware of a similar association between calnexin and lipogenesis.

### The profile of *BiP* expression in cultured cells is distinct in high-altitude deer mice

Adaptation at high altitudes involves reprogramming of the animals' metabolic program to satisfy, among other demands, the elevated demands for thermogenesis and insulation. Therefore, we explored whether the differences in the UPR profile recorded in *P. maniculatus* have ramifications in the adaptation to different environments. Analysis of animals from a colony established in the past from ancestors captured at high altitudes (SM2 population) (Vrana et al., 2014) showed that, in general, these animals exhibited more intense UPR than the low-altitude population (BW animals), as evaluated by the number of genes that are in the top 50th, 25th or 5th percentile of the combined BW and SM2 population (Fig. 5). For this analysis, the percentage of animals in each of the SM2 or BW populations that have at least 1 UPR gene in the highest 50th percentile (Fig. 5A), that have all 4 UPR genes in the highest 25th percentile (Fig. 5B), or that have 3 or 4 UPR genes in the highest 5th percentile (Fig. 5C), of the total population are shown. The corresponding distribution for the number of genes in each analysis is shown in Fig. 5D, E and F, respectively.

The hypothesis that these differences are due to the distinct genetic makeup of the SM2 and BW stocks is also supported by the fact that a preliminary analysis of the promoter of *BiP* showed that the 2 stocks have polymorphic *BiP* promoter alleles. Analysis of ∼40 randomly selected animals from the BW stock showed the presence of a predominant allele with A at position −603 (from TATA box), G at position −519, G at position −477, G at position −392, A at position −176 and C at position −161. In the minor allele, the corresponding nucleotides had been substituted by G,C,A,C,C,G, respectively, all linked. In the SM2 population, besides the predominant allele, additional alleles had been discovered in 40 randomly selected individuals. Those alleles had G,G,G,G,C,G or G,G,G,G,A,G or G,G,G,G,C,C or A,G,G,G,C,G in the corresponding positions, but not the minor allele discovered in the BW stock.

In view of the positive correlation between UPR genes and plasma lipids, a testable hypothesis is that high-altitude animals had elevated plasma levels of lipids. Indeed, the levels of LDL, and to a lesser extent of total cholesterol (but not of HDL), were elevated both prior to and after high-fat-diet administration (Fig. 5G). It is plausible that enhanced UPR expression represents an adaptation that facilitates enhanced lipogenesis at high-altitude populations that, in turn, may satisfy the increased needs for thermogenesis and insulation in rodents (Vaillancourt et al., 2009; Cheviron et al., 2012) and humans (Temte, 1996; Hirschler et al., 2012; Mohanna et al., 2006).

### The UPR profile *ex vivo* predicts hepatic steatosis induced by high-fat diet

**Fig. 6.**
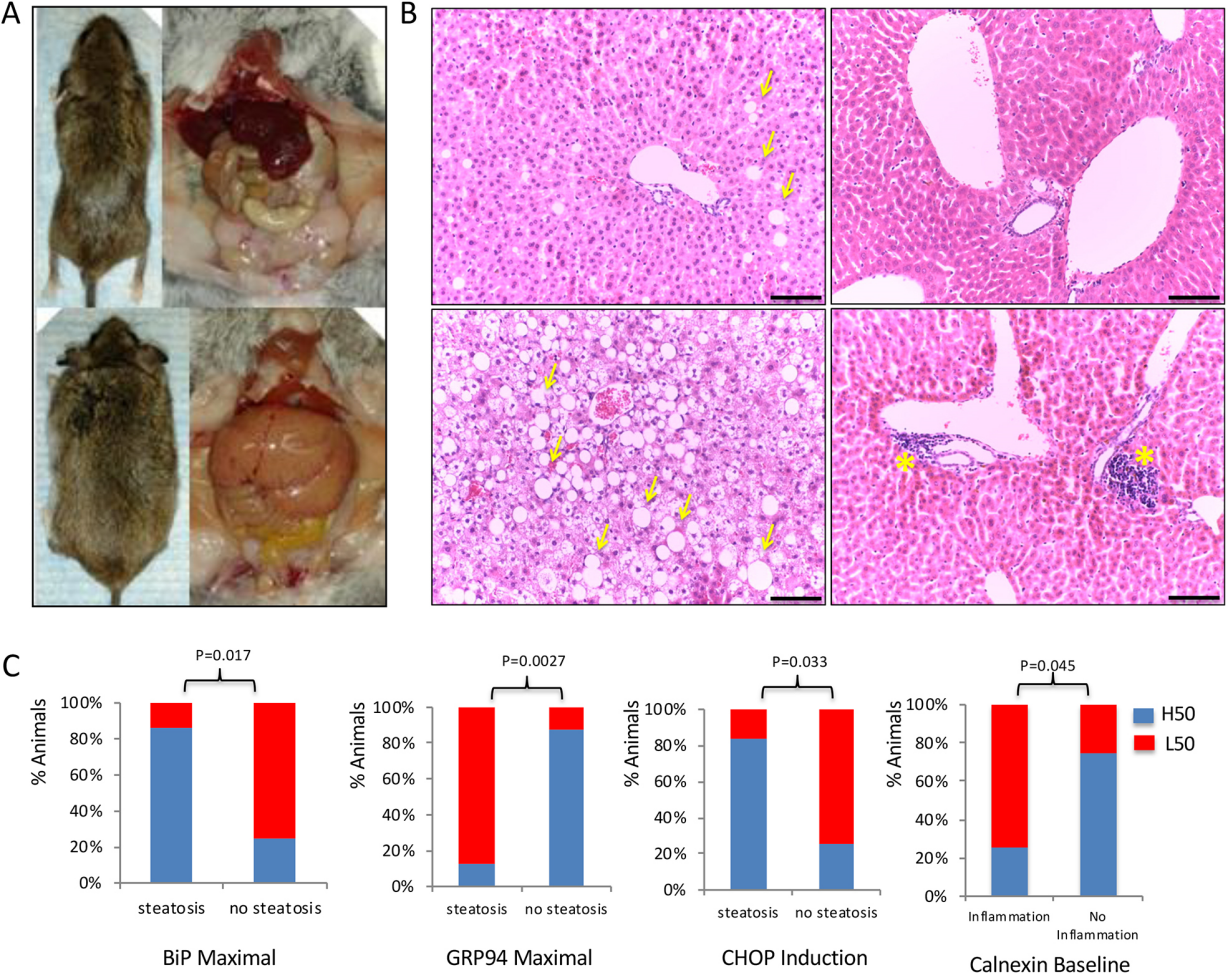
**Long-term administration of high-fat diet causes metabolic pathologies at variable degrees in *P. maniculatus* (*n*=16).** (A) Macroscopic and necropsy images showing animals with minimal (top) or high (bottom) fat accumulation after administration of high-fat/sucrose diet for 6 months. (B) H&E-stained sections of livers from mice showing minimal or no evidence of pathology (top row), high degree of hepatic steatosis (bottom left) or portal inflammation (bottom right). Yellow arrows indicate cells accumulating high amounts of fat; yellow asterisks indicate portal inflammation. Scale bar: 100 μm. (C) Correlation between steatosis (recorded in 8 of 16 animals), or portal inflammation (recorded in 8 of 16 animals), and whether the expression of the corresponding UPR-related gene was assigned to the top 50th (H50) or bottom 50th (L50) percentile in the animals tested. *P*-values (chi-square test) are indicated.

We subsequently tested whether the profile of UPR *ex vivo* predicts the onset of chronic diseases, such as hepatic steatosis, that are caused by long-term administration of a high-fat/sucrose diet. Therefore, a cohort of animals, aged 3 months, for which the UPR profile had been recorded at puberty, were subjected to a high-fat/sucrose diet for 6 months and analyzed for evidence of fatty liver disease. In view of the fact that UPR expression is generally coordinated, this analysis involved animals exhibiting low coordination to facilitate assessment of the individual genes' predictive value. Although none developed diabetes, elevated baseline insulin levels, indicative of the onset of insulin resistance, were positively associated with high baseline levels of *GRP94* (not shown). Histopathological analysis upon termination of the study showed that 50% of the animals exhibited steatosis, occasionally associated with portal inflammation of variable degrees (Fig. 6A,B) that was correlated with the baseline levels of calnexin in the fibroblasts (*P*=0.045) (Fig. 6C). No lobar inflammation, ballooning degeneration of the hepatocytes or fibrosis of the stroma was observed. Analysis of steatosis onset, in concert with *GRP94* expression *ex vivo*, identified the latter as a protective factor for disease development. The majority of the animals with steatosis (87.5%) had maximal *GRP94* levels in culture at the lower 50th percentile as opposed to only 12.5% of the animals that developed steatosis and had maximal *GRP94* at the higher 50th percentile (*P*=0.0027, chi-square test) (Fig. 6C). It is to be noted that both baseline and maximal levels of *GRP94* exhibited positive correlation with HDL and total cholesterol – but not LDL – levels prior to high-fat-diet administration, but this association was abolished after 2 weeks of a high-fat/sucrose diet. As opposed to *GRP94*, which was protective, high maximal *BiP* expression (*P*=0.017) in fibroblasts and inducibility of *CHOP* (*P*=0.033) were predictive, for higher sensitivity to steatosis (Fig. 6). To that end, the increased incidence of steatosis in the animals with higher *BiP* expression could be the trade-off for the enhanced lipogenesis that may be beneficial at high altitudes.

## DISCUSSION

Although the linearity in the expression of UPR-associated genes has been studied extensively by induced qualitative changes in gene expression, whether the response to ER stress exhibits coordination and variability among individuals remains inadequately studied. In the present study, we used genetically diverse deer mice as a model to record the profile of the UPR in cultured primary cells isolated at puberty. Our analyses showed that UPR induction at the mRNA level was highly variable between different animals, yet a high degree of coordination was maintained in the expression levels of individual targets. To that end, some animals exhibited a more intense, while others a milder, response to ER stress. Implications of this in the adaptation of animals at different environments are suggested by the recorded tendency of high-altitude-originating deer mice to exhibit a more intense UPR. This observation became apparent regardless of the cut-off of the gene number considered. It is noted, though, that this association may reflect founder effects during the establishment of SM2 colonies of *P. maniculatus*, and that wild-caught animals should also be used to validate and exploit this finding. However, the presence of different alleles with regards to polymorphisms in the *BiP* promoter between the 2 different stocks supports the notion that the populations are genetically distinct and that certain differences in the regulatory regions of UPR genes, such as *BiP*, may contribute to the distinct profile of the UPR in these stocks.

Whether UPR variation accounted here in culture reflects varying efficiency for protein folding or augmented response during challenge remains unknown. Although neither possibility can be excluded formally, our data are mostly aligned with the latter possibility. We postulate that if the differences recorded here were primarily due to the inherent, varied ability of the individuals for protein folding, then no association between baseline expression and maximal expression would have been recorded, because the baseline levels would be assumed to be different but the maximal levels should have remained similar. In addition, fibroblasts were analyzed *in vitro*, after propagation in the Petri dish, decreasing the possibility that systemic factors might have caused the differences recorded. Formally, the possibility that systemic factors might have caused epigenetic changes in the fibroblasts cannot be ruled out, but this appears rather unlikely.

The availability of individual animals with variable response to UPR allowed us to explore whether inherent variation in UPR is associated with deregulation of lipid homeostasis, a condition that is highly relevant to ER stress. Thus, lipid levels were assessed in animals prior to and after high-fat-diet administration. It is noted that although isoflurane is known to induce hyperglycemia, this is unlikely to affect lipid levels, yet, isoflurane was applied to the control groups as well. It is emphasized that the administration of a special diet occurred in adults, after the characterization of the fibroblasts that occurred at puberty. Our results suggest that the UPR profile of cultured fibroblasts isolated early in life is linked to the onset of metabolic pathologies at the organismal level. With regards to the lipid levels in the plasma, the *ex vivo* levels of all 4 genes tested showed independently a positive association with the baseline plasma lipid levels, underscoring the link between ER stress and lipid metabolism. After high-fat-diet administration, the association persisted only for *CHOP* and calnexin, implying the engagement of these UPR genes, particularly after diet-induced stress.

Prolonged administration of high-fat diet and induction of steatosis in a subset of the animals facilitated assessment of the predictive value of individual UPR-associated genes in disease development. This considerable variation in steatosis development is seen only in the outbred *Peromyscus* used here, because, in laboratory mice, prolonged administration of high-fat diet, albeit variably among different inbred strains, induces steatosis rather uniformly within the animals of the same strain, while some variation exists only in the portion of the liver exhibiting evidence of disease (VanSaun et al., 2009; Parks et al., 2013; Fontana et al., 2013; Norheim et al., 2017).

Animals for which UPR genes, especially *BiP* and *GRP94*, are not coordinated were subjected to a high-fat diet, thereby appreciating the individual gene's contribution. Analysis showed that the UPR profile in fibroblasts early in life predicted the onset of diet-induced hepatic steatosis. An intriguing finding was the protective role of *GRP94* against steatosis despite the positive association with lipid levels in the plasma, implying the operation of a predictive factor capable of dissociating hyperlipidemia with hepatic disease. For GRP94, a role in suppressing liver cancer has been proposed (Chen et al., 2014b,c), but no direct involvement of GRP94 in steatosis is available, which may point to a novel action of this chaperone in lipid metabolism.

With regards to *BiP*, the present analysis showed that higher *BiP* expression in fibroblasts was associated with increased risk for steatosis. It is noteworthy that it is the specific deletion of *BiP* in hepatocytes that promotes steatosis in laboratory mice (*Mus*), implying a protective role for BiP in disease development (Ji et al., 2011; Chen et al., 2014a). It is conceivable that this controversy reflects the pleiotropy of BiP function and its cumulative role in lipid metabolism beyond the liver. Enhanced expression of BiP in the liver may thus be protective, as pointed out by the mouse studies. However, this benefit is counteracted by its presumably negative role in other peripheral tissues. Consistent with this observation, systemic administration of chemical chaperones alleviates hepatic steatosis in rodent models of the disease (Ozcan et al., 2006). Furthermore, the transcription factor XBP1 that is activated during the UPR has been shown to possess lipogenic activity (Chen et al., 2014c), even though an anti-lipogenic role has also been shown (Fontana et al., 2013). Another plausible explanation is that the genetic ablation of *BiP* *in vivo* produces phenotypes that may be qualitatively distinct from the reduction of BiP expression that occurs physiologically in the genetically diverse experimental animals used here. The cascade of events triggered by the deletion of *BiP* and other UPR-associated genes in mice may cause phenotypic manifestations that are not highly relevant to the naturally occurring versions of hypomorphic *BiP* alleles seen in deer mice. This may also explain the apparent discrepancy that, depending on the model, both lipogenic and antilipogenic activities for the same UPR gene may be revealed (Lee et al., 2008; Herrema et al., 2016).

We found calnexin's involvement to be intriguing. Although it exhibited a similar predictive activity to *CHOP* for lipidemia, no association with steatosis was recorded. It is likely that these observations reflect shared regulation between CHOP and calnexin, or a direct regulatory association between them during metabolic stress.

Whether UPR variation at the level of the mRNA recorded here also reflects similar variation at the protein level remains to be established. Unfortunately, the absence of *Peromyscus*-specific antibodies or antibodies that adequately work on this genus to allow quantification of protein levels precludes this analysis at this point. In addition, we note that mRNA levels as assessed here should rather be viewed as indicators of differential activation of the UPR. Protein levels may directly follow, or not, the mRNA levels; however, an additional level of complexity will be introduced, that of post-translational modification, which might have masked the correlation between UPR gene expression and pathophysiological parameters. As such, assessing protein levels may be useful for mechanistic studies addressing the role of individual chaperones, yet activation of the UPR could not be monitored accurately by evaluating protein levels.

Different ER-stress-linked genes were generally associated with distinct parameters, either biochemical or histological, but, depending on the specific gene-pathology combination, it can be the baseline, induction or maximal levels that would have the higher value. It is likely that, while baseline levels reflect a chronic stress state, exemplified by *GRP94* and *BiP* levels, inducibility and especially maximal levels reflect acute stress and are exemplified by all 4 ER stress-related genes analyzed here. The power of the protein-processing machinery to operate as a modifier of disease outcomes has been shown recently by the demonstration that the activity of *HSP90* (also known as *HSP90AA1*), the cytosolic paralog of *GRP94*, determined the severity of the cancer predisposition syndrome Fanconi anemia (Taipale et al., 2014).

Collectively, the ability of the UPR profile to predict the outcome of ER-stress-associated pathologies later in life shows that the UPR intensity reflects a systemic response rather than a reaction of isolated organs and tissues. It is conceivable that, by appropriate selection of chaperones or combinations, the onset of different ER-stress-related pathologies can be predicted. It is likely that, by using this platform, consisting of different stressors in outbred populations, unforeseen links between specific ER-stress-associated genes and various diseases could be revealed. Finally, the role of inherent variation of the UPR in the adaptation to different environments needs to be elucidated.

## MATERIALS AND METHODS

### Fibroblast culture and tunicamycin treatment

Fibroblasts were isolated from ear punches collected during routine weaning and marking procedures. Ear punches were washed for 2 min in 70% EtOH and moved to complete RPMI medium. Ear punches were processed by cutting and treating in collagenase I (at 100 U/ml) for 1 h. Tissue was removed from the culture once cells were visible. Cells were cultured in RPMI-1640+10% fetal bovine serum+500 U/ml penicillin+500 µl/ml streptomycin+0.292 mg/ml L-glutamine and passed when cells were at 90% confluency or above to 45% confluency or above. Cells were passed no more than 4 times before tunicamycin treatment unless otherwise described. For tunicamycin treatment, cells were split into 6-well plates, at 300,000 cells/well, and left for 24 h. After 24 h, tunicamycin was added to the medium at a concentration of 5 µg/ml for 5 h, immediately followed by RNA extraction.

### Cholesterol measurement

Blood was collected from animals via submandibular puncture, once before high-fat-diet administration and once after. Blood samples were frozen at −80°C prior to use. Cholesterol was measured using an HDL and LDL/VLDL Cholesterol Assay Kit (Abcam). The procedure was followed as provided with the kit, without modification.

### Animals

Deer mice (*P. maniculatus*) were obtained at the time of weaning from the Peromyscus Genetic Stock Center (PGSC), University of South Carolina (USC), Columbia, SC (RRID:SCR_002769). Treatment beyond fibroblast collection was not started until animals were aged 3-4 months. Animals were placed on a high-fat diet (58% kcal/fat with sucrose, Research Diets D12331) for 2 weeks or 6 months. After the high-fat diet, animals were sacrificed using isoflurane as an anesthetic followed by cervical dislocation, and the pancreas, liver, kidneys, muscle and adipose tissue were collected. Tissues for RNA were frozen using dry ice. All experiments were approved by the Institutional Animal Care and Use Committee of the USC.

### RNA extraction, cDNA synthesis and qPCR

RNA extraction was performed using an RNeasy Mini Kit (Qiagen), using the supplied protocol, with modifications. Spin time for the wash steps was increased from 15 s to 30 s. The extra spin step prior to elution was included. RNA was eluted using 30 μl nuclease-free water. Complementary DNA (cDNA) synthesis was performed using an iScript cDNA synthesis kit (Bio-Rad) according to the supplied protocol. Quantitative PCR (qPCR) was performed on a T100 thermocycler (Bio-Rad) using iTaq Universal SYBR Green Supermix (Bio-Rad). Oligonucleotide sequences used for qPCR amplification were designed using Primer3 (Untergasser et al., 2012) and PrimerBLAST and are shown in Table 1. Oligonucleotides for the amplification of the *BiP* promoter are shown in Table 1; 982 bp were amplified and sequenced.

**Table 1.**
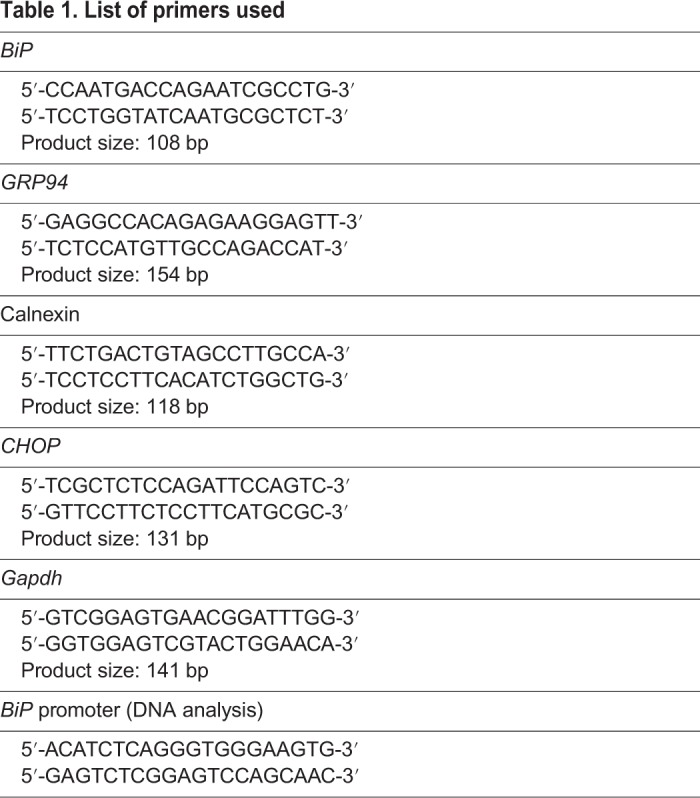
**List of primers used**

### Histology

At sacrifice, the liver was excised from each animal, fixed for 1-2 days in 4% formaldehyde, dehydrated with 100% EtOH, and embedded in wax. Paraffin sections were stained with Hematoxylin and Eosin (H&E). Histological examination of the liver specimens was performed blindly for the presence of nonalcoholic fatty liver disease (NAFLD) according to the scoring system designed by the Pathology Committee of the NASH Clinical Research Network, which addresses the full spectrum of lesions of NAFLD (Kleiner et al., 2005). Images shown were obtained by a Leica ICC50 HD.

### Statistical analysis

Results were analyzed using 2-tailed Student's *t*-test or Pearson's correlation. *P*<0.05 was considered significant.

## Supplementary Material

Supplementary information
