# Supplementary material for: Differential regulation of the unfolded protein response in outbred deer mice and susceptibility to metabolic disease
Source: Dis Model Mech. 2019 Feb 27;12(2):dmm037242. doi: 10.1242/dmm.037242 (PMC6398494; doi:10.1242/dmm.037242)
Supplement: Supplementary information [file dmm-12-037242-s1.pdf]

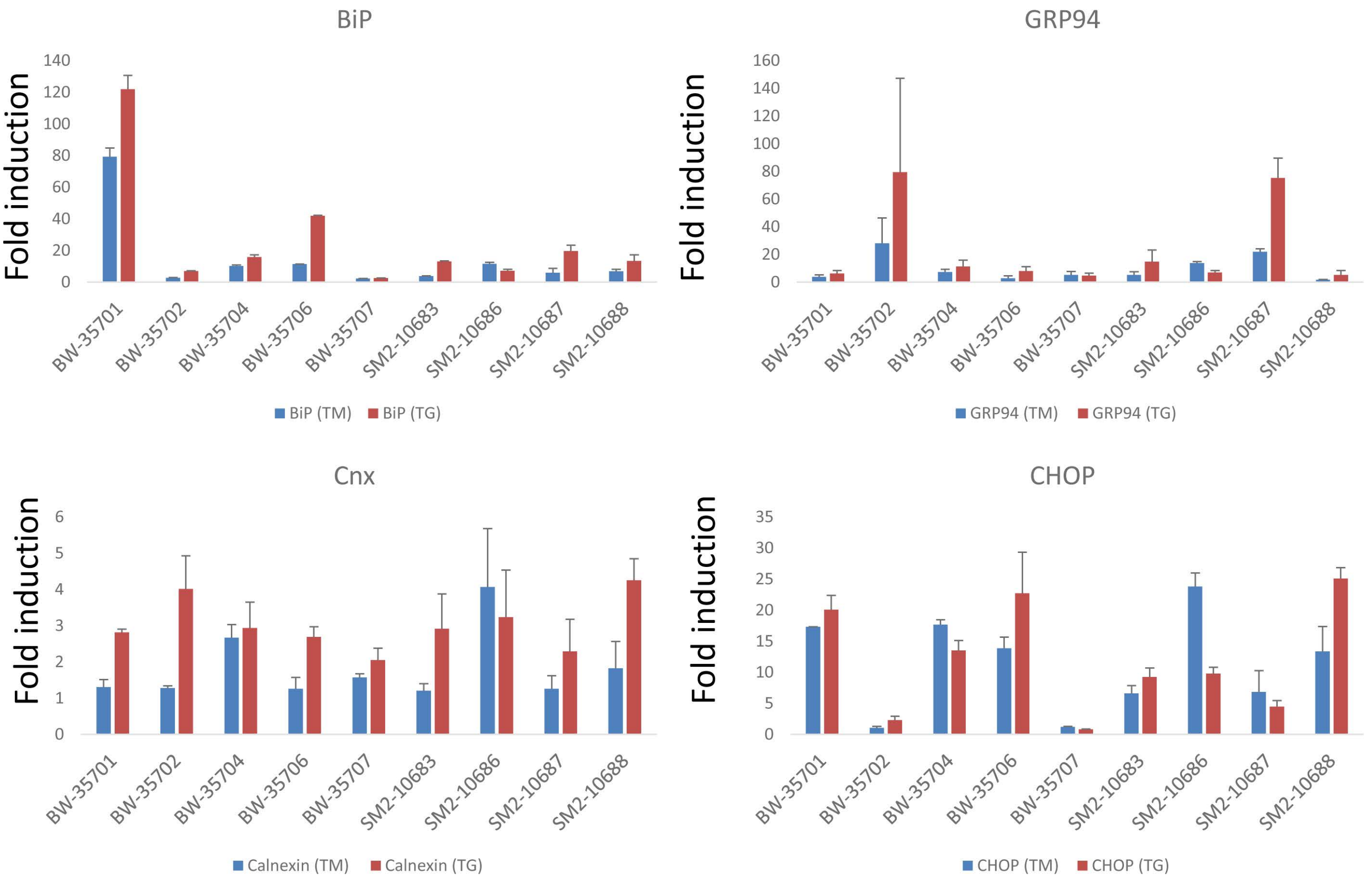

Figure S1. The expression of BiP, GRP94, Cnx and CHOP in fibroblasts from different animals exposed to Tunicamycin (TM) or Thapsigargin (TG). Exposure to TG was performed at 1µM for 5h. Vertical bars indicate SEM from 2 replicas. Animal number is indicated.

Disease Models & Mechanisms • Supplementary information

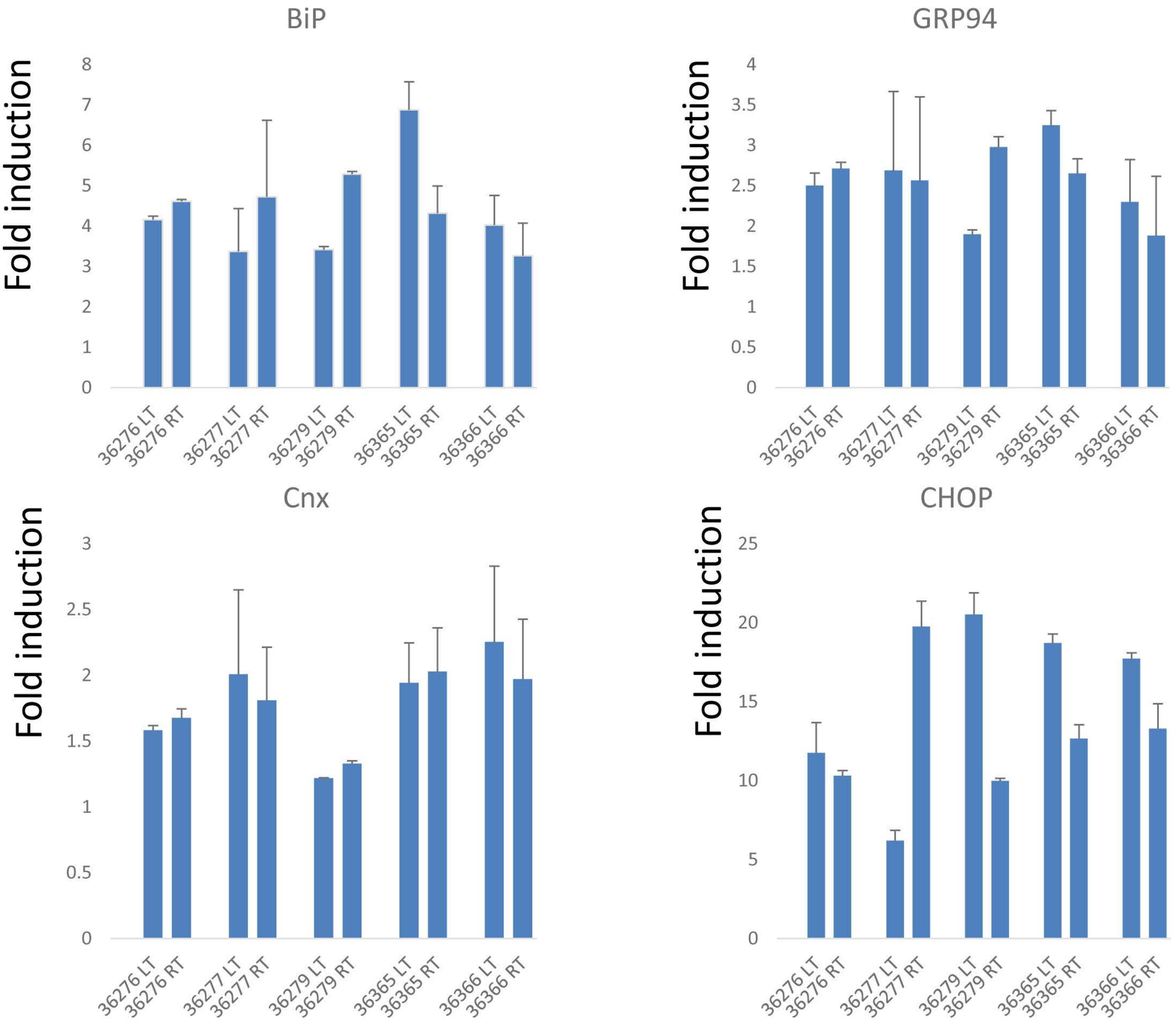

Figure S2. UPR expression profile in different fibroblast preparation isolated from the left (LT) or right (RT) ear of different animals. Vertical bars indicate SEM from 2 replicas. Animal number is indicated.

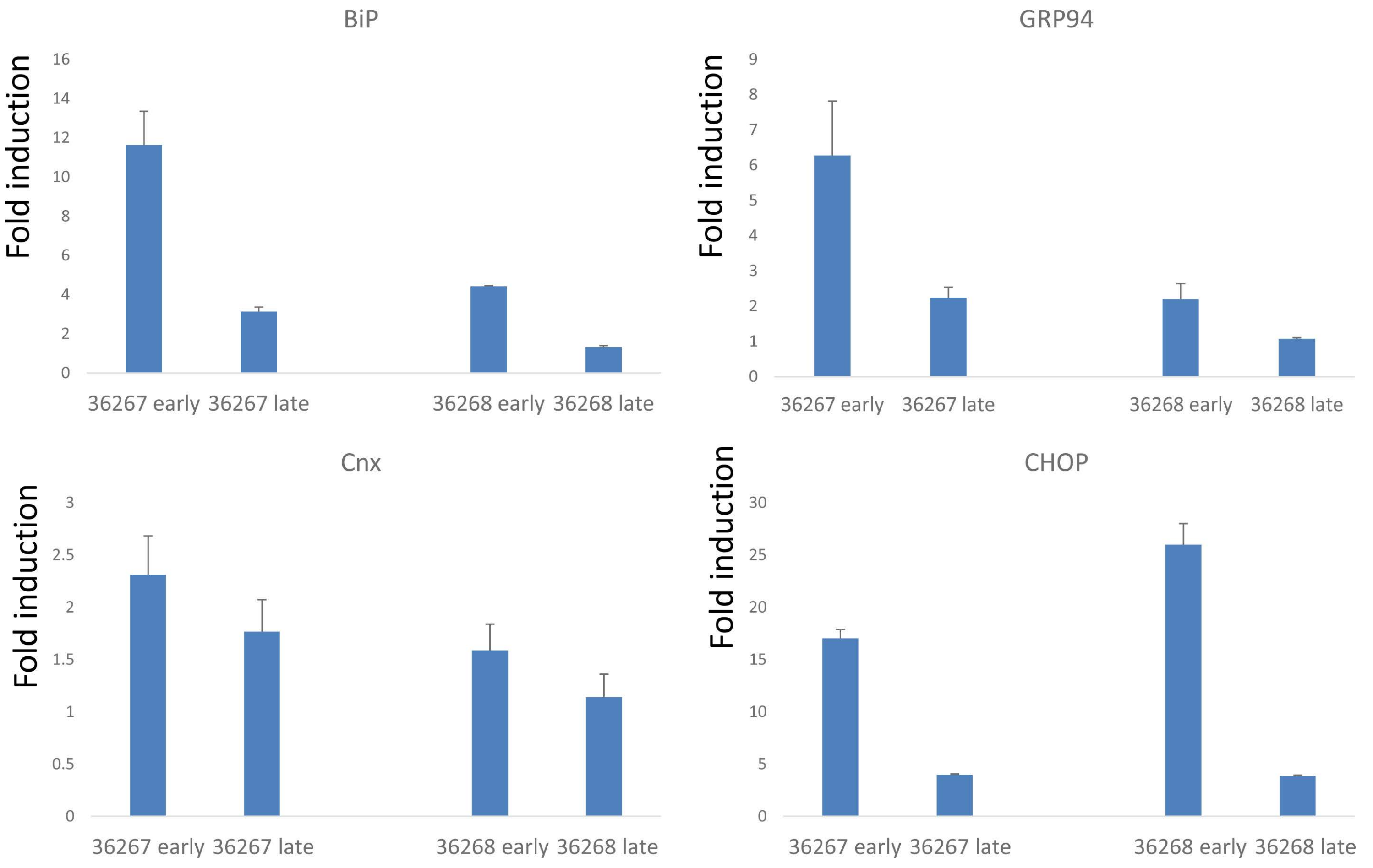

Figure S3. Comparison in the expression profile of BiP, GRP94, CNX and CHOP in fibroblasts analyzed at early (2-3) or late (5-6) passage. Vertical bars indicate SEM from 2 replicas. Animal number is indicated.

Disease Models & Mechanisms • Supplementary information
